# Neuromodulation of Aerobic Exercise—A Review

**DOI:** 10.3389/fpsyg.2015.01890

**Published:** 2016-01-07

**Authors:** Saskia Heijnen, Bernhard Hommel, Armin Kibele, Lorenza S. Colzato

**Affiliations:** ^1^Cognitive Psychology Unit, Leiden UniversityLeiden, Netherlands; ^2^Leiden Institute for Brain and Cognition, Leiden UniversityLeiden, Netherlands; ^3^Institute for Sports and Sport Science, University of KasselKassel, Germany

**Keywords:** endocannabinoids, dopamine, serotonin, BDNF, stress, aerobic exercise, HPA axis

## Abstract

Running, and aerobic exercise in general, is a physical activity that increasingly many people engage in but that also has become popular as a topic for scientific research. Here we review the available studies investigating whether and to which degree aerobic exercise modulates hormones, amino acids, and neurotransmitters levels. In general, it seems that factors such as genes, gender, training status, and hormonal status need to be taken into account to gain a better understanding of the neuromodular underpinnings of aerobic exercise. More research using longitudinal studies and considering individual differences is necessary to determine actual benefits. We suggest that, in order to succeed, aerobic exercise programs should include optimal periodization, prevent overtraining and be tailored to interindividual differences, including neuro-developmental and genetically-based factors.

## Introduction

Aerobic exercise is known to activate the body's stress response, the hypothalamic-pituitary-adrenal (HPA) axis, and yet many people engage in sports like running because they perceive its effects as relaxing. Solving this apparent contradiction calls for a distinction between “good stress” and “bad stress,” or physical and psychological stress respectively (for ease of discussion), which in turn requires a deeper analysis of the neural mechanisms underlying both stress and aerobic exercise. Whereas stress research traditionally takes frequent runners as models for understanding chronic stress (Duclos et al., 1998), clinical studies are discovering the potential for exercise to alleviate depression and anxiety (Marco et al., 2011). Both acute and chronic exercise may even lead to improved cognitive performance (see Hillman et al., 2008; Chang et al., 2012, for a comprehensive review; Colzato et al., 2013), whereas psychological stress is known to impair cognitive functions (Colzato et al., 2008; see Lupien et al., 2009, for a comprehensive review). In the present review article we provide a summary of the available studies and their main results on the neuromodular underpinnings of aerobic exercise to gain a better understanding of what makes exercise-induced stress different from stress due to negative life events. We will elaborate on how aerobic exercise influences cortisol, endocannabinoids (eCBs), brain-derived neurotrophic factor (BDNF), dopamine, and serotonin and discuss a number of interindividual factors that influence the effects. In doing so, we hope to inform future studies of important factors that should be considered when designing and analyzing experiments and training programs involving aerobic exercise, so that issues that are thus far unresolved may be studied in an effective way.

## The neuroendocrine response

The physical stress response starts with the hypothalamus secreting corticotrophin-releasing hormone, which travels straight to the anterior pituitary to induce adrenocorticotropic hormone release into the general circulation (Ranabir and Reetu, 2011). Once this reaches the adrenal cortex, cortisol is released into the bloodstream. This has inhibitory effects upon the hypothalamus and pituitary (Crosby and Bains, 2012) through medial prefrontal cortex (mPFC) receptors (Hill et al., 2011) and reduces stress-induced overexcitability of the amygdala (Gray et al., 2015). Completion of this circuit restores the homeostasis and is a sign of a healthy stress-response.

The psychological (bad) stress response is largely the same, but whereas physical (good) stress is accompanied by an increase in growth hormone, psychological stress rarely is (Ranabir and Reetu, 2011). Prolonged psychological stress may lead to major depression (Miller and O'Callaghan, 2002), and in this population the salivary cortisol response to negative events is blunted, with men having a more blunted response than women (Peeters et al., 2003).

Running leads to elevated levels of cortisol lasting at least 2 h after exercising, as measured in healthy men (mean age = 41 years) from saliva and plasma (Duclos et al., 1998; Labsy et al., 2013). The aerobic exercise requires an intensity of at least 60% of VO2max (maximum capacity of oxygen uptake) to elicit a reliable cortisol response (Labsy et al., 2013).

The stress response adapts, indicated by the finding that following a rest day, cortisol levels of frequent endurance exercisers upon awakening are similar to those of the sedentary, both in urine and in saliva (Gouarné et al., 2005; Labsy et al., 2013). The inactivation of cortisol into cortisone increases proportionally to the amount of exercise (and so to cortisol increase, Gouarné et al., 2005). However, this is not the case for chronic psychological stress, which leads to an increased cortisol response upon awakening, without increased inactivation of cortisol (Wüst et al., 2000a,b). Hence, what makes physical stress different from psychological stress is the increased inactivation of the active steroid (cortisol) into the inert steroid (cortisone). This mechanism is crucial because it protects trained individuals against the deleterious effects of prolonged increased cortisol secretion (Gouarné et al., 2005), which include hypertension, hyperglycemia (Whitworth et al., 2005), major depressive episode and anorexia nervosa (Ehlert et al., 2001).

## Endocannabinoids

The positive mood after aerobic exercise, and running specifically, has traditionally been ascribed to endorphins. The first human evidence of increased central endorphin levels comes from a positron emission tomography study in which 10 athletes were scanned at rest and after 2 h of running. The level of euphoria (as indexed by subjective ratings) was significantly increased after running and was inversely correlated with opioid binding in frontolimbic brain areas (Boecker et al., 2008). However, debate surrounds the endorphin hypothesis (Kolata, 2002; Sparling et al., 2003) and it has been shown in mice that blocking eCB receptors, but not endorphin receptors, diminishes the anxiolytic and analgesic effects of running (Fuss et al., 2015). Additionally, there are indications that eCBs actually cause the release of endorphins in the hypothalamus (Bakkali-Kassemi et al., 2011). Researchers have thus recently turned their attention to eCBs (Sparling et al., 2003). eCBs are synthesized both centrally and peripherally, on-demand, by a post-synaptic stimulated neuron, and travel to the presynaptic neuron where they bind to the cannabinoid receptor type 1 (CB1R; Crosby and Bains, 2012). A single session of endurance training at 70–80% of the maximum heart rate capacity provides the optimal eCB increase (Raichlen et al., 2013), demonstrated in a four-day intervention in which healthy, regularly running men ran at varying degrees of intensity (50/70/80/90% of maximal capacity) in a randomized order. There is a high density of CB1Rs in the frontal cortex, amygdala, hippocampus, and the hypothalamus (Marco et al., 2011), areas crucial for emotional homeostasis.

One eCB has been implicated in psychological and physical stress: anandamide (AEA). AEA seems responsible for tonic responses (Gunduz-Cinar et al., 2013) and it increases slowly upon cortisol stimulation, and normalizes amygdalar hyperactivity (Ferreira-Vieira et al., 2014; Gray et al., 2015). These two processes are glucocorticoid-dependent and slowly normalize the stress response (±30 min) after physical exercise. AEA, a fatty chemical that easily crosses the blood–brain barrier, seems critical in supporting the beneficial effects of aerobic exercise on mood. Given that AEA concentration decreases upon psychological stress (Gunduz-Cinar et al., 2013) but increases after physical exercise (Heyman et al., 2012), AEA may be considered a key neurochemical in the clinical potential of exercise in alleviating depression and anxiety through its regulatory role in amygdala hyperactivity (Marco et al., 2011).

Whereas aerobic exercise leads to increased eCB levels and hippocampal CB1R density in rats (Hill et al., 2010), prolonged psychological stress leads to an increase in the enzyme that breaks anandamide down (fatty acid amide hydrolase; Reich et al., 2009). The divergence between the two effects may explain the differential moods following the two types of stress.

## Brain-derived neurotrophic factor

There is evidence suggesting that AEA increase during exercise might be one of the key elements in the exercise-induced increment in BDNF levels and that high AEA levels during recovery might postpone the return of BDNF to baseline (Heyman et al., 2012). BDNF exerts beneficial effects on cognition through its ability to enhance neurogenesis, synaptic plasticity and long-term potentiation, the basis of learning (Leckie et al., 2014). Interestingly, BDNF increase after physical exercise is positively correlated with cognitive performance (age range = 19–28 years, Tsai et al., 2014) and AEA increase in the brain seems to be the mediator for the cognitive benefits exerted by BDNF. Specifically, blocking the CB1Rs in rat brains has been shown to diminish the BDNF increase following exercise (Ferreira-Vieira et al., 2014), demonstrating the a link between AEA binding and BDNF increase.

Both acute and chronic psychological stress have been shown to reduce BDNF levels in rats, though less robustly upon chronic stress (Murakami et al., 2005). Decreased serum BDNF levels have been reported in mood disorders and by people under great psychological stress (Martinowich and Lu, 2008).

The BDNF increase may differ across exercise regimen as well as gender. A recent review (Huang et al., 2014) concludes that a single session of aerobic exercise (running or cycling, ranging from 20 to 90 min of 40–75% of maximal power output or 40–60% of VO2max or 75% of maximal heart rate) increases BDNF levels and that frequent aerobic training (running, or walking for elderly populations, cycling, ranging from 45 min three times a week for 12 weeks, to 60 min five times a week for 6 months, and a range of 60–90% of VO2max, 50–75% of heart rate reserve) magnifies this increase. Interestingly, in a meta-analysis of aerobic exercise (running, cycling rowing, swimming at ranges from 60 to 95% VO2max) and BDNF with similar results, studies with a greater proportion of females had lower effect sizes (Szuhany et al., 2015), suggesting a less robust BDNF increase for women. We speculate that this may due to the fact that the HPA axis interacts with hormonal changes across the menstrual cycle and that increases in progesterone levels increment AEA breakdown (Maccarrone et al., 2001). Consistent with this idea, when training at 70% of VO2max, adrenocorticotropic hormone, vasopressin and glucose are significantly more increased during the mid-luteal phase compared with the follicular phase in healthy women (Altemus et al., 2001). Moreover, higher adrenocorticotropic hormone and glucose levels suggest that cortisol levels might be higher in the mid-luteal phase in response to exercise (Altemus et al., 2001). Accordingly, given the link between sexual hormones and cognitive functions (Colzato et al., 2010; Colzato and Hommel, 2014), we hypothesize that aerobic exercise might affect females' mood and cognition differently in the follicular phase (low progesterone levels) than in the mid-luteal phase (high progesterone levels). Specifically, during the luteal phase the exercise-induced increase in AEA will rapidly break down, which in turn will decrease the amount of BDNF released. A prediction from this assumption would be that engagement in aerobic exercise during the luteal phase will deteriorate cognitive benefits compared to the follicular phase.

In contrast to psychological stress, which is known to impair cognitive functions across the lifespan (Lupien et al., 2009), a seminal recent study has shown that in a healthy elderly population (55–80 years old), moderate intensity walking (60% VO2max, 40 min, 52 weeks) increased serum BDNF levels. This increment translated to a constant (not improved) task switching performance, whereas their sedentary peers' performance declined (Leckie et al., 2014). Remarkably, exercise not only serves to improve cognitive function, but it also might help keep it intact in a population undergoing a decrease in BDNF (Tapia-Arancibia et al., 2008).

Interestingly, the Val66Met BDNF gene that plays a role in sensitivity to anxiety and cortisol stress response (Colzato et al., 2011) as well as baseline BDNF levels, is also implicated in motivation to exercise (Hooper et al., 2014). Specifically, people with at least one Met allele on the Val66Met BDNF gene have lower BDNF expression, smaller hippocampal volume and relatively low performance on memory tasks. These same people (mean age studied = 23 years) appear to have a more positive mood response to acute, moderate intensity exercise (Hooper et al., 2014). This result is particularly interesting in suggesting that exercise may compensate for unfavorable genetic predispositions by reducing individual differences in memory functions. Furthermore, whereas Val homozygotes show a constant level of intrinsic motivation to exercise, people with at least one Met allele showed an increase of intrinsic motivation after an endurance training session (Hooper et al., 2014). In addition, they were more likely to continue exercising when they were told they could stop if they wanted. This points to an inverted u-shaped function where people with low baseline levels of BDNF seem to benefit relatively more from exercise as it pushes their BDNF levels up in the direction of the optimum. Based on this idea, we suggest that the efficiency of aerobic training programs and the beneficial effect on cognition might be modulated by interindividual differences, including pre-existing neuro-developmental factors and genetic differences. Consequently, training programs that are tailored to individual abilities, skills, and needs might be more likely to succeed.

So whereas psychological stress decreases BDNF levels, aerobic exercise leads to an increment which is modulated by gender, genes and age. This aids in explaining divergent memory and neuroplasticity effects following the two types of stress.

## Neurotransmitters: serotonin and dopamine

Muscle activity requires uptake of branched-chain amino acids. These are normally in competition with tryptophan, the precursor of serotonin, to be carried across the blood–brain barrier. By reducing the amount of competitive amino acids through muscle uptake, aerobic exercise increases tryptophan's chances of crossing the blood–brain barrier, and so has the potential to increase serotonin in the brain (Patrick and Ames, 2015). Serotonin is an important neurotransmitter for emotional processing (Harmer, 2008) and serves memory functions in the hippocampus (Haider et al., 2006).

The changes in brain serotonin metabolism are time-course dependent and differ between brain regions. The striatum, hippocampus and mid-brain of rodents show an elevation of serotonin and serotonin turnover following acute exercise (Dey et al., 1992; Meeusen and De Meirleir, 1995). Additionally, frequent exercise (30 min of swimming daily, 4 weeks) increased serotonin synthesis and metabolism in the cerebral cortex and brain stem (Dey et al., 1992; Meeusen and De Meirleir, 1995), but decreased serotonin levels in the hippocampus directly after training (Dey et al., 1992). The cortical changes lasted at least a week after discontinuing exercise, whereas serotonin activity in the brainstem is diminished by that time (Dey et al., 1992). The hypothalamus shows decreased serotonin metabolism one day after training, increasing again after a week's rest (Dey et al., 1992). As serotonin induces release of corticotrophin-releasing hormone (Dey et al., 1992), this decrease may serve to reduce the stress response during prolonged exercise.

Upon chronic and acute psychological (bad) stress, tryptophan hydroxylase levels have been shown to be upregulated in rat brains, enabling increased serotonin synthesis (Murakami et al., 2005). This supports earlier findings that chronic stress leads to higher serotonin levels in the hippocampus, hypothalamus, frontal cortex and striatum, though the latter two may only show upregulation in males (Adell et al., 1988). This is likely a consequence of decreased serotonin transporter levels in the raphe nuclei following both acute and prolonged stress (Martinowich and Lu, 2008). The serotonin transporter is responsible for transporting serotonin from the synaptic cleft to the presynaptic neuron, terminating its stimulation. There is research linking serotonin receptors to the BDNF decrease seen after prolonged stress. Specifically, activation of the 5-HT2a serotonin receptor decreased hippocampal BDNF levels in rats (Vaidya et al., 1999). So increased serotonin levels following prolonged bad stress may be responsible for the BDNF decrease associated with depression.

With regard to dopamine, which is important in working memory, mental flexibility and early stages of motor control (Nieoullon, 2002), there appears to be a divergent pattern of the effect of psychological stress for males and females. Frontal cortex dopamine levels have been found to be increased in female, but decreased in male rats following stress (Bowman et al., 2003). Accordingly, female rats demonstrated improved performance on memory tasks, whereas male rats demonstrated an impairment. By contrast, physical stress has been shown to lead to an eCB-induced dopamine boost from the nucleus accumbens (young, well-trained cyclists: Fattore et al., 2008; Heyman et al., 2012). In addition, aerobic exercise has been shown to increase dopamine levels in the striatum, hypothalamus, midbrain, and brainstem in various animal studies (Foley and Fleshner, 2008), further supporting the beneficial effects of exercise on memory and mood.

Increases in serotonin and dopamine seem to modulate fatigue upon prolonged exercise. Exhaustion appears to set in when dopamine levels start to drop while serotonin levels are still elevated (Meeusen and De Meirleir, 1995). The serotonin agonist quipazine dimaleate (acting on the 5-HT3 receptor) appears to shorten time to exhaustion and withholds the exercise-induced dopamine boost normally occurring after around 1 h of running in tested animals. The serotonin receptor antagonist LY53857 can increase time to exhaustion and prevents the dopamine decrease (Meeusen and De Meirleir, 1995). This fact may explain why runners usually take food supplements based on tyrosine, the precursor of dopamine, during endurance training, given that it could prevent the decrease of dopamine. Not surprisingly, several studies have shown that the intake of tyrosine may compensate cognitive decline associated with cognitive challenges (Colzato et al., 2014; Steenbergen et al., 2015) and stress (Deijen and Orbleke, 1994).

Again, a divergent pattern following the two types of stress is evident. Psychological stress increases serotonin synthesis, possibly to the point of depletion, whereas dopamine undergoes gender-specific changes. By contrast, aerobic exercise increases dopamine in both genders, lowers baseline serotonin levels in the nigrostriatal tract and down-regulates certain receptor subtypes.

## Conclusion

All in all, it seems that what makes exercise-induced stress different from stress due to negative life events is that, in contrast to psychological (bad) stress, physical (good) stress is associated with increased inactivation of cortisol (active steroid) into cortisone (inert steroid), increased level of AEA, BDNF, and serotonin. In particular, it seems that aerobic exercise modulates hormone, neurotrophin and neurotransmitter levels depending on factors such as genes, age and hormonal status. Accordingly, future studies need to consider individual differences more systematically. Indeed, if aerobic exercise really affects neuroplasticity, it is possible that the effect of aerobic exercise on performance depends on the pre-experimental performance level of the individual. Addressing this issue will require (more) longitudinal studies. For any future research, a standardized hormone sampling procedure (response upon awakening, Wüst et al., 2000b; but also before and after exercise, Kanaley et al., 2001) and exercise protocol (70–80% of maximum capacity based on age-adjusted maximum heart rate for eCB research, Raichlen et al., 2013; minimum of 60% of maximal heart rate for cortisol research, Labsy et al., 2013) would be advised for ease of comparison between studies. Importantly, aerobic exercise programs should include optimal periodization and prevent overtraining (Angeli et al., 2004). Moreover, since efficacy of aerobic exercise may depend on various factors, among which is genetic variability related to BDNF, we suggest taking into account such inter-individual variability and tailoring exercise to individual needs will be crucial for developing successful endurance programs in the future.

## Funding

This work was supported by research grant from the Netherlands Organization for Scientific Research (NWO) awarded to LC (Vidi grant: #452-12-001).

### Conflict of interest statement

The authors declare that the research was conducted in the absence of any commercial or financial relationships that could be construed as a potential conflict of interest.

## References

[B1] Adell A. Garcia-Marquez C. Armario A. Gelpi E. (1988). Chronic stress increases serotonin and noradrenaline in rat brain and sensitizes their responses to a further acute stress. J. Neurochem. 50, 1678–1681. 10.1111/j.1471-4159.1988.tb02462.x 2453609

[B2] Altemus M. Roca C. Galliven E. Romanos C. Deuster P. (2001). Increased vasopressin and adrenocorticotropin responses to stress in the midluteal phase of the menstrual cycle. J. Clin. Endocrinol. Metab. 86, 2525–2530. 10.1210/jcem.86.6.7596 11397850

[B3] Angeli A. Minetto M. Dovio A. Pacotti P. (2004). The overtraining syndrome in athletes: a stress-related disorder. J. Endocrinol. Invest. 27, 603–612. 10.1007/BF03347487 15717662

[B4] Bakkali-Kassemi L. El Ouezzani S. Magoul R. Merroun I. Lopez-Jurado M. Errami M. (2011). Effects of cannabinoids on neuropeptide Y and β-endorphin expression in the rat hypothalamic arcuate nucleus. Br. J. Nutr. 105, 654–660. 10.1017/S0007114510004095 21134330

[B5] Boecker H. Sprenger T. Spilker M. E. Henriksen G. Koppenhoefer M. Wagner K. J.. (2008). The runner's high: opioidergic mechanisms in the human brain. Cereb. Cortex 18, 2523–2531. 10.1093/cercor/bhn013 18296435

[B6] Bowman R. E. Beck K. D. Luine V. N. (2003). Chronic stress effects on memory: sex differences in performance and monoaminergic activity. Horm. Behav. 43, 48–59. 10.1016/S0018-506X(02)00022-3 12614634

[B7] Chang Y. K. Labban J. D. Gapin J. I. Etnier J. L. (2012). The effects of acute exercise on cognitive performance: a meta-analysis. Brain Res. 1453, 87–101. 10.1016/j.brainres.2012.02.068 22480735

[B8] Colzato L. S. Hertsig G. van den Wildenberg W. P. M. Hommel B. (2010). Estrogen modulates inhibitory control in healthy human females: evidence from the stop-signal paradigm. Neuroscience 167, 709–715. 10.1016/j.neuroscience.2010.02.029 20219635

[B9] Colzato L. S. Hommel B. (2014). Effects of estrogen on higher-order cognitive functions in unstressed human females may depend on individual variation in dopamine baseline levels. Front. Neurosci. 8:65. 10.3389/fnins.2014.00065 24778605 PMC3985021

[B10] Colzato L. S. Jongkees B. J. Sellaro R. van den Wildenberg W. P. M. Hommel B. (2014). Eating to stop: tyrosine supplementation enhances inhibitory control but not response execution. Neuropsychologia 62, 398–402. 10.1016/j.neuropsychologia.2013.12.027 24433977

[B11] Colzato L. S. Kool W. Hommel B. (2008). Stress modulation of visuomotor binding. Neuropsychologia 46, 1542–1548. 10.1016/j.neuropsychologia.2008.01.006 18295287

[B12] Colzato L. S. Szapora A. Pannekoek J. N. Hommel B. (2013). The impact of physical exercise on convergent and divergent thinking. Front. Hum. Neurosci. 7:824. 10.3389/fnhum.2013.00824 24348370 PMC3845014

[B13] Colzato L. S. van der Does A. J. W. Kouwenhoven C. Elzinga B. M. Hommel B. (2011). BDNF Val66Met polymorphism is associated with higher anticipatory cortisol stress response, anxiety, and alcohol consumption in healthy adults. Psychoneuroendocrinology 36, 1562–1569. 10.1016/j.psyneuen.2011.04.010 21596481

[B14] Crosby K. M. Bains J. S. (2012). The intricate link between glucocorticoids and endocannabinoids at stress-relevant synapses in the hypothalamus. Neuroscience 204, 31–37. 10.1016/j.neuroscience.2011.11.049 22155492

[B15] Deijen J. B. Orbleke J. F. (1994). Effect of tyrosine on cognitive function and blood pressure under stress. Brain Res. Bull. 33, 319–323. 10.1016/0361-9230(94)90200-3 8293316

[B16] Dey S. Singh R. H. Dey P. K. (1992). Exercise training: significance of regional alterations in serotonin metabolism of rat brain in relation to antidepressant effect of exercise. Physiol. Behav. 52, 1095–1099. 10.1016/0031-9384(92)90465-E 1283013

[B17] Duclos M. Corcuff J. B. Arsac L. Moreau-Gaudry F. Rashedi M. Roger P.. (1998). Corticotroph axis sensitivity after exercise in endurance-trained athletes. Clin. Endocrinol. (Oxf.) 48, 493–501. 10.1046/j.1365-2265.1998.00334.x 9640417

[B18] Ehlert U. Gaab J. Heinrichs M. (2001). Psychoneuroendocrinological contributions to the etiology of depression, posttraumatic stress disorder, and stress-related bodily disorders: the role of the hypothalamus-pituitary-adrenal axis. Biol. Psychol. 57, 141–152. 10.1016/S0301-0511(01)00092-8 11454437

[B19] Fattore L. Fadda P. Spano M. S. Pistis M. Fratta W. (2008). Neurobiological mechanisms of cannabinoid addiction. Mol. Cell. Endocrinol. 286, S97–S107. 10.1016/j.mce.2008.02.006 18372102

[B20] Ferreira-Vieira T. H. Bastos C. P. Pereira G. S. Moreira F. A. Massensini A. R. (2014). A role for the endocannabinoid system in exercise- induced spatial memory enhancement in mice. Hippocampus 24, 79–88. 10.1002/hipo.22206 24115292

[B21] Foley T. E. Fleshner M. (2008). Neuroplasticity of dopamine circuits after exercise: implications for central fatigue. Neuromolecular Med. 10, 67–80. 10.1007/s12017-008-8032-3 18274707

[B22] Fuss J. Steinle J. Bindila L. Auer M. K. Kirchherr H. Lutz B.. (2015). A runner's high depends on cannabinoid receptors in mice. Proc. Natl. Acad. Sci. U.S.A. 112, 13105–13108. 10.1073/pnas.1514996112 26438875 PMC4620874

[B23] Gouarné C. Groussard C. Gratas-Delamarche A. Delamarche P. Duclos M. (2005). Overnight urinary cortisol and cortisone add insights into adaptation to training. Med. Sci. Sports Exerc. 37, 1157–1167. 10.1249/01.mss.0000170099.10038.3b 16015133

[B24] Gray J. M. Vecchiarelli H. A. Morena M. Lee T. T. Hermanson D. J. Kim A. B.. (2015). Corticotropin-releasing hormone drives anandamide hydrolysis in the amygdala to promote anxiety. J. Neurosci. 35, 3879–3892. 10.1523/JNEUROSCI.2737-14.2015 25740517 PMC4348185

[B25] Gunduz-Cinar O. Hill M. N. McEwen B. S. Holmes A. (2013). Amygdala FAAH and anandamide: mediating protection and recovery from stress. Trends Pharmacol. Sci. 34, 637–644. 10.1016/j.tips.2013.08.008 24325918 PMC4169112

[B26] Haider S. Khaliq S. Ahmed S. P. Haleem D. J. (2006). Long-term tryptophan administration enhances cognitive performance and increases 5HT metabolism in the hippocampus of female rats. Amino Acids 31, 421–425. 10.1007/s00726-005-0310-x 16699826

[B27] Harmer C. J. (2008). Serotonin and emotional processing: does it help explain antidepressant drug action? Neuropharmacology 55, 1023–1028. 10.1016/j.neuropharm.2008.06.036 18634807

[B28] Heyman E. Gamelin F. X. Goekint M. Piscitelli F. Roelands B. Leclair E.. (2012). Intense exercise increases circulating endocannabinoid and BDNF levels in humans—possible implications for reward and depression. Psychoneuroendocrinology 37, 844–851. 10.1016/j.psyneuen.2011.09.017 22029953

[B29] Hill M. N. McLaughlin R. J. Pan B. Fitzgerald M. L. Roberts C. J. Lee T. T. Y.. (2011). Recruitment of prefrontal cortical endocannabinoid signaling by glucocorticoids contributes to termination of the stress response. J. Neurosci. 31, 10506–10515. 10.1523/JNEUROSCI.0496-11.2011 21775596 PMC3179266

[B30] Hill M. N. Titterness A. K. Morrish A. C. Carrier E. J. Lee T. T. Y. Gil- Mohapel J.. (2010). Endogenous cannabinoid signaling is required for voluntary exercise-induced enhancement of progenitor cell proliferation in the hippocampus. Hippocampus 20, 513–523. 10.1002/hipo.20647 19489006 PMC2847038

[B31] Hillman C. H. Erickson K. I. Kramer A. F. (2008). Be smart, exercise your heart: exercise effects on brain and cognition. Nat. Rev. Neurosci. 9, 58–65. 10.1038/nrn2298 18094706

[B32] Hooper A. E. C. Bryan A. D. Hagger M. S. (2014). What keeps a body moving? The brain-derived neurotrophic factor val66met polymorphism and intrinsic motivation to exercise in humans. J. Behav. Med. 37, 1180–1192. 10.1007/s10865-014-9567-4 24805993

[B33] Huang T. Larsen K. T. Ried-Larsen M. Møller N. C. Andersen L. B. (2014). The effects of physical activity and exercise on brain-derived neurotrophic factor in healthy humans: a review. Scand. J. Med. Sci. Sports 24, 1–10. 10.1111/sms.12069 23600729

[B34] Kanaley J. A. Weltman J. Y. Pieper K. S. Weltman A. Hartman M. L. (2001). Cortisol and growth hormone responses to exercise at different times of day. J. Clin. Endocrinol. Metab. 86, 2881–2889. 10.1210/jc.86.6.2881 11397904

[B35] Kolata G. (2002). Runner's high? Endorphins? Fiction say some scientists. The New York Times, 21 May.

[B36] Labsy Z. Prieur F. Le Panse B. Do M. C. Gagey O. Lasne F.. (2013). The diurnal patterns of cortisol and dehydroepiandrosterone in relation to intense aerobic exercise in recreationally trained soccer players. Stress 16, 261–265. 10.3109/10253890.2012.707259 22734443

[B37] Leckie R. L. Oberlin L. E. Voss M. W. Prakash R. S. Szabo-Reed A. Chaddock- Heyman L.. (2014). BDNF mediates improvements in executive function following a 1-year exercise intervention. Front. Hum. Neurosci. 8:985. 10.3389/fnhum.2014.00985 25566019 PMC4263078

[B38] Lupien S. J. McEwen B. S. Gunnar M. R. Heim C. (2009). Effects of stress throughout the lifespan on the brain, behaviour and cognition. Nat. Rev. Neurosci. 10, 434–445. 10.1038/nrn2639 19401723

[B39] Maccarrone M. Valensise H. Bari M. Lazzarin N. Romanini C. Finazzi- Agrò A. (2001). Progesterone up-regulates anandamide hydrolase in human lymphocytes: role of cytokines and implications for fertility. J. Immunol. 166, 7183–7189. 10.4049/jimmunol.166.12.7183 11390466

[B40] Marco E. M. García-Gutiérrez M. S. Bermúdez-Silva F. J. Moreira F. A. Guimarães F. Manzanares J.. (2011). Endocannabinoid system and psychiatry: in search of a neurobiological basis for detrimental and potential therapeutic effects. Front. Behav. Neurosci. 5:63. 10.3389/fnbeh.2011.00063 22007164 PMC3186912

[B41] Martinowich K. Lu B. (2008). Interaction between BDNF and serotonin: role in mood disorders. Neuropsychopharmacology 33, 73–83. 10.1038/sj.npp.1301571 17882234

[B42] Meeusen R. De Meirleir K. (1995). Exercise and brain neurotransmission. Sports Med. 20, 160–188. 10.2165/00007256-199520030-00004 8571000

[B43] Miller D. B. O'Callaghan J. P. (2002). Neuroendocrine aspects of the response to stress. Metabolism 51, 5–10. 10.1053/meta.2002.33184 12040534

[B44] Murakami S. Imbe H. Morikawa Y. Kubo C. Senba E. (2005). Chronic stress, as well as acute stress, reduces BDNF mRNA expression in the rat hippocampus but less robustly. Neurosci. Res. 53, 129–139. 10.1016/j.neures.2005.06.008 16024125

[B45] Nieoullon A. (2002). Dopamine and the regulation of cognition and attention. Prog. Neurobiol. 67, 53–83. 10.1016/S0301-0082(02)00011-4 12126656

[B46] Patrick R. P. Ames B. N. (2015). Vitamin D and the omega-3 fatty acids control serotonin synthesis and action, part 2: relevance for ADHD, bipolar, schizophrenia, and impulsive behavior. FASEB J. 29, 2207–2222. 10.1096/fj.14-268342 25713056

[B47] Peeters F. Nicholson N. A. Berkhof J. (2003). Cortisol responses to daily events in major depressive disorder. Psychosom. Med. 65, 836–841. 10.1097/01.PSY.0000088594.17747.2E 14508029

[B48] Ranabir S. Reetu K. (2011). Stress and hormones. Indian J. Endocrinol. Metab. 15, 18. 10.4103/2230-8210.77573 21584161 PMC3079864

[B49] Raichlen D. A. Foster A. D. Seillier A. Giuffrida A. Gerdeman G. L. (2013). Exercise-induced endocannabinoid signaling is modulated by intensity. Eur. J. Appl. Physiol. 113, 869–875. 10.1007/s00421-012-2495-5 22990628

[B50] Reich C. G. Taylor M. E. McCarthy M. M. (2009). Differential effects of chronic unpredictable stress on hippocampal CB1 receptors in male and female rats. Behav. Brain Res. 203, 264–269. 10.1016/j.bbr.2009.05.013 19460405 PMC2747651

[B51] Sparling P. B. Giuffrida A. Piomelli D. Rosskopf L. Dietrich A. (2003). Exercise activates the endocannabinoid system. Cogn. Neurosci. Neuropsychol. 17, 2209–2211. 10.1097/00001756-200312020-00015 14625449

[B52] Steenbergen L. Sellaro R. Hommel B. Colzato L. S. (2015). Tyrosine promotes cognitive flexibility: evidence from proactive vs. reactive control during task switching performance. Neuropsychologia 69, 50–55. 10.1016/j.neuropsychologia.2015.01.022 25598314

[B53] Szuhany K. L. Bugatti M. Otto M. W. (2015). A meta-analytic review of the effects of exercise on brain-derived neurotrophic factor. J. Psychiatr. Res. 60, 56–64. 10.1016/j.jpsychires.2014.10.003 25455510 PMC4314337

[B54] Tapia-Arancibia L. Aliaga E. Silhol M. Arancibia S. (2008). New insights into brain BDNF function in normal aging and Alzheimer disease. Brain Res. Rev. 59, 201–220. 10.1016/j.brainresrev.2008.07.007 18708092

[B55] Tsai C. L. Chen F. C. Pan C. Y. Wang C. H. Huang T. H. Chen T. C. (2014). Impact of acute aerobic exercise and cardiorespiratory fitness on visuospatial attention performance and serum BDNF levels. Psychoneuroendocrinology 41, 121–131. 10.1016/j.psyneuen.2013.12.014 24495613

[B56] Vaidya V. A. Terwilliger R. M. Z. Duman R. S. (1999). Role of 5- HT 2A receptors in the stress-induced down-regulation of brain-derived neurotrophic factor expression in rat hippocampus. Neurosci. Lett. 262, 1–4. 10.1016/S0304-3940(99)00006-3 10076858

[B57] Whitworth J. A. Williamson P. M. Mangos G. Kelly J. J. (2005). Cardiovascular consequences of cortisol excess. Vasc. Health Risk Manag. 1, 291. 10.2147/vhrm.2005.1.4.291 17315601 PMC1993964

[B58] Wüst S. Federenko I. Hellhammer D. H. Kirschbaum C. (2000a). Genetic factors, perceived chronic stress, and the free cortisol response to awakening. Psychoneuroendocrinology 25, 707–720. 10.1016/S0306-4530(00)00021-4 10938450

[B59] Wüst S. Wolf J. Hellhammer D. H. Federenko I. Schommer N. Kirschbaum C. (2000b). The cortisol awakening response—normal values and confounds. Noise Health 2, 79. 12689474

